# Re-Introduction of Transmembrane Serine Residues Reduce the Minimum Pore Diameter of Channelrhodopsin-2

**DOI:** 10.1371/journal.pone.0050018

**Published:** 2012-11-19

**Authors:** Ryan Richards, Robert E. Dempski

**Affiliations:** Department of Chemistry and Biochemistry, Worcester Polytechnic Institute, Worcester, Massachusetts, United States of America; University of Toronto, Canada

## Abstract

Channelrhodopsin-2 (ChR2) is a microbial-type rhodopsin found in the green algae *Chlamydomonas reinhardtii*. Under physiological conditions, ChR2 is an inwardly rectifying cation channel that permeates a wide range of mono- and divalent cations. Although this protein shares a high sequence homology with other microbial-type rhodopsins, which are ion pumps, ChR2 is an ion channel. A sequence alignment of ChR2 with bacteriorhodopsin, a proton pump, reveals that ChR2 lacks specific motifs and residues, such as serine and threonine, known to contribute to non-covalent interactions within transmembrane domains. We hypothesized that reintroduction of the eight transmembrane serine residues present in bacteriorhodopsin, but not in ChR2, will restrict the conformational flexibility and reduce the pore diameter of ChR2. In this work, eight single serine mutations were created at homologous positions in ChR2. Additionally, an endogenous transmembrane serine was replaced with alanine. We measured kinetics, changes in reversal potential, and permeability ratios in different alkali metal solutions using two-electrode voltage clamp. Applying excluded volume theory, we calculated the minimum pore diameter of ChR2 constructs. An analysis of the results from our experiments show that reintroducing serine residues into the transmembrane domain of ChR2 can restrict the minimum pore diameter through inter- and intrahelical hydrogen bonds while the removal of a transmembrane serine results in a larger pore diameter. Therefore, multiple positions along the intracellular side of the transmembrane domains contribute to the cation permeability of ChR2.

## Introduction

Channelrhodopsin-2 (ChR2) is a light-activated cation channel found in the eyespot region of the green algae *Chlamydomonas reinhardtii* [1]. Together with channelrhodopsin-1, ChR2 helps mediate light-sensing and phototaxis [1]–[4] in *C. reinhardtii*. ChR2 is activated upon photoisomerization of the chromophore all-trans retinal with blue light [5], [6]. Like other microbial-type rhodopsins, ChR2 is comprised of seven transmembrane domains [4], [7], [8] with a retinal moiety bound to a single lysine residue [9]. However, unlike other rhodopsins, ChR2 is able to conduct a large array of mono- and divalent cations with a high selectivity for protons [4]. Large cations such as guanidinium (ionic radius ∼2.4 Å) [10] and dimethyl-amine (ionic radius ∼2.6 Å) [10] can pass through the channel which suggests a large pore diameter [4], [11]. Under continuous illumination, ChR2 current decays from an initial peak current to stationary current. The stationary current then decays biexponentially to a desensitized closed state once the light is turned off [4], [12], [13].

**Figure 1:**
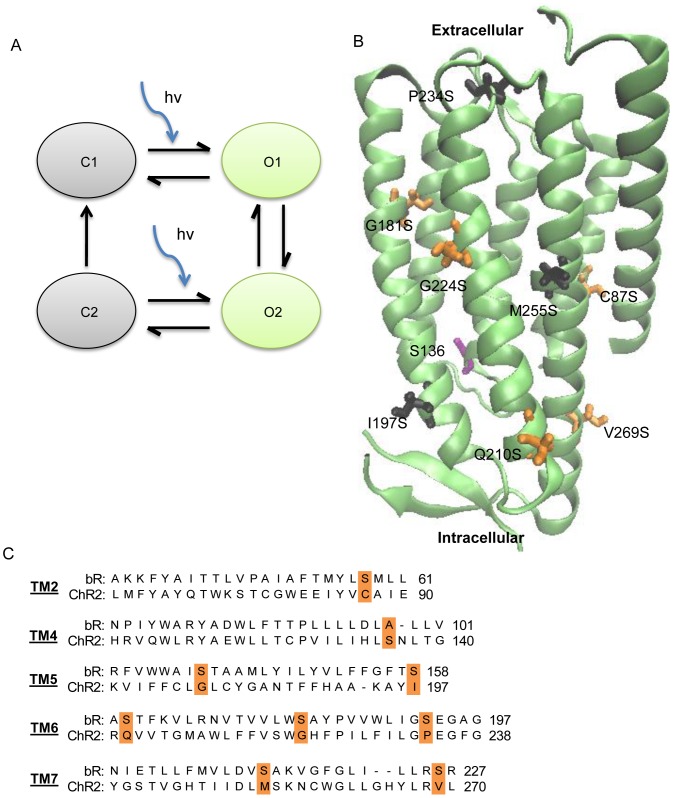
Simplified photocycle and ChR2 single serine mutation model. (A) Simple four-state model of the channelrhodopsin-2 photocycle. The two nonconducting states consist of a dark-adapted (C1) and desensitized (C2) state. The conducting states represent an early conducting state (O1) and a late conducting state (O2). Blue arrows represent illumination with blue light. (B) Structural model of ChR2 based on the channelrhodopsin chimera C1C2 (PDB entry: 3UG9) highlighting the locations of serine mutations. Three residues (black) had highly reduced photocurrents, while 5 mutations showed changes in pore size, permeability, and/or kinetics (orange). Residue S136 is shown in purple. The retinal chromophore, which is covalently bound to K257, is shown in yellow. This figure was prepared using Visual Molecular Dynamics [37]. (C) Sequence comparison of transmembrane domains between bacteriorhodopsin and channelrhodopsin-2. Residues that correspond to serine mutations are highlighted in orange. Adapted from [9].

The photocycle of ChR2 has been described by both a three-state [4], [14], [15] and four-state model [4], [7], [12], each of which contains several spectral intermediates as identified by time-resolved UV-Vis spectroscopy [16]. The three-state model can accurately predict the peak to stationary current in addition to the fast kinetic values associated with the rise and decay rates; however it fails to provide a quantitative description of the biphasic decay after the light is turned off [13]. In contrast, the four-state model can quantitatively describe the experimental values obtained from photocurrent decay with both fast and slow time constants (Figure 1A) [12]–[14].

ChR2 shares a high sequence homology with other microbial type rhodopsins, but in contrast with these proteins, ChR2 functions mainly as a cation channel and not an ion pump. However, recent studies have shown that when ChR2 is reconstituted in planar lipid membranes in the absence of an electrochemical gradient, it functions as an outwardly rectified proton pump [17]. Thus, it has been proposed that ChR2 is a proton pump with an inherent leak current that exhibits ion channel properties [17].

ChR2 also undergoes large conformational changes when compared to other opsin proteins [18]. These structural changes have been attributed to the backbone movement of the channel through FTIR and Raman difference spectroscopy [18]. These changes, which occur before and after the conductive state, suggest that pore formation precedes gating upon activation of the channel. Furthermore, an analysis of the sequence of ChR2 reveals that this protein lacks specific residues which contribute to non-covalent forces in bacteriorhodopsin (bR). These residues have been shown to play a role in the hydrogen-bonding network and overall stability of bR [8], [19], [20].

Non-covalent forces, such as inter- and intrahelical hydrogen bonding [21]–[23] and helix-helix interactions [24], [25] are critical for proper folding [25], insertion [23], and function [8], [19] of membrane proteins. Specifically, serine and threonine residues have been shown to be involved in helical packing interactions and motifs [23]. Furthermore, a high percentage of serine and threonine residues found within the transmembrane domains of membrane proteins form hydrogen bonds with carbonyl oxygen atoms in the backbone structure [21] or other polar amino acids [19]. These hydrogen bonds stabilize protein structures and can be involved in ion transport across the cell membrane.

A sequence alignment of bR and ChR2 reveals that there are eight serine residues within the transmembrane domains of bR that are not present in ChR2. Previously [20], these residues in bR were individually mutated to Ala in order to disrupt the hydrogen-bonding and proton transfer network. It was shown that some, but not all, serine mutations had altered proton pumping ability, kinetics, and chromophore regeneration. Additionally, all eight mutants were functional pumps. These experiments elucidated specific residues that contribute to the stability and structural changes of bR through hydrogen-bonding, but are not essential for proton transport.

The importance of hydrogen-bonding between specific residues has also been observed in ChR2 [7], [16]. Mutations located at C128 have been shown to increase the conduction lifetime of ChR2 [26]. C128, located on helix 3, has been proposed to form a hydrogen bond with D156 on helix 4 and is important for proper channel function. This hydrogen bond pair is consistent throughout most microbial-type rhodopsins [27]. In bR, the pair corresponds to T90 and D115 where its contribution to protein stability has been studied previously [20], [28]. Neither residue is directly involved in the proton conduction pathway, but disruption of this hydrogen-bond alters the kinetics and proton pumping ability. However, the recent crystal structure of the channelrhodopsin chimera C1C2 shows that the distance between the thiol group of C167 and carboxyl oxygen of D196, which correspond to C128 and D156 in ChR2, is 4.4 Å, too large for hydrogen-bonding [29]. Instead, it was suggested that the thiol group interacts with the conjugated region of the retinal moiety [29].

Why ChR2 undergoes much larger structural changes compared to other opsin proteins is still unknown. However, ChR2 lacks specific residues and motifs that contribute to inter- and intrahelical interactions that are present in other microbial-type rhodopsins. We hypothesize that reintroduction of the eight transmembrane serine residues present in bR, but not in ChR2, will restrict the conformational flexibility and reduce the pore diameter of ChR2. In our present work, we created single serine ChR2 constructs that correspond to homologous residues in bR. We used two-electrode voltage clamp techniques on *Xenopus laevis* oocytes to study channel permeability, kinetics, and pore size in varying alkali metal solutions. ChR2 has an estimated pore size [4] larger than voltage gated sodium channels (∼4.7 Å) [30] but smaller than the nicotinic acetylcholine receptor (∼8.4 Å) [10]. Minimum pore sizes can be determined using excluded volume theory [10], [31] and can help elucidate specific residues involved in forming the narrowest region of the pore. Three substitutions, I197S, P234S, and M255S, resulted in largely reduced photocurrents compared to wild-type ChR2. Furthermore, G224S showed changes in permeability and kinetics while C87S, V269S, G181S and Q210S showed changes in permeability and pore size with no effect on kinetics. Lastly, S136A resulted in a larger minimum pore diameter. These results indicate the importance of hydrogen-bonding on the ChR2 pore size, conformational flexibility, and ion selectivity. An analysis of our experiments demonstrates that the size selectivity filter for ion conductance is not located at a single region within the transmembrane domains. Instead determinants that contribute to ion conductance are distributed along the intracellular side of channelrhodopsin-2.

## Materials and Methods

### Ethical Animal Treatment

Isolation of *Xenopus laevis* oocytes were performed in strict accordance to the guidelines given in the Guide for the Care and Use of Laboratory Animals of the National Institutes of Health. The surgical protocol was approved by the WPI Institutional Animal Care and Use Committee (protocol number- A3277–01). Surgery was performed using the anesthetic tricaine to minimize animal suffering.

### Reagents

The mMessage mMachine SP6 kit was purchased from Ambion, Inc. (Austin, TX). Restriction enzymes were acquired from New England Biolabs, Inc. (Ipswich, MA). The Quikchange site-directed mutagenesis kit was bought from Stratagene. (La Jolla, CA). All other reagents were purchased from Sigma-Aldrich Corp. (St. Louis, MO) unless otherwise noted.

### Molecular Biology

A truncated wild type channelrhodopsin-2 (ChR2, amino acids 1–309) with the hemagluttanin (HA) epitope sequence (YPYDVPDYA) was unidirectionally cloned into the vector pTLN [32] using *EcoRV* and *XbaI* restriction sites. Serine mutations were made using the Quikchange site-directed mutagenesis kit. All mutations were verified by full gene sequencing.

### Oocyte Preparation and mRNA Synthesis

Oocytes were isolated according to previously published protocols [33]. In brief, oocytes were obtained via collagenase digestion (3 mg/mL; 3 hours, 17°C, shaking) in ORI^+^ solution (90 mM NaCl, 2 mM KCl, 2 mM CaCl_2_, 5 mM MOPS; pH 7.4) after partial ovarectomy of *Xenopus laevis* frogs. ChR2 mRNA was prepared using the SP6 mMessage and mMachine kit. A volume of 50 nL of 1 µg/µL mRNA was injected into each oocyte and incubated in ORI^+^ containing 1 mg/ml gentamycin and 1 µM all-trans-retinal (1 mM stock in DMSO) in the dark at 17°C for 3–4 days.

### Oocyte Membrane Preparation and Western Blotting

*Xenopus* oocyte total membrane fractions were prepared as described previously [34], [35]. Briefly, oocytes were homogenized in 20 µL/oocyte homogenization buffer A (20 mM Tris, 5 mM MgCl_2,_ 5 mM NaH_2_PO_4_, 1 mM EDTA, and 80 mM sucrose; pH 7.4) supplemented with 1 mM PMSF and 5 µg/ml leupeptin and pepstatin A, Homogenized oocytes were spun at 200 g for 5 minutes at 4°C. After removal of supernatant, centrifugation was repeated. The supernatant was removed and spun at 14,000 g for 20 minutes at 4°C to pellet the membrane. Protein samples were solubilized in 4 µl/oocyte Laemmli buffer and incubated at 37°C for 30 minutes for denaturing.

Protein samples were separated on a 12% gel using SDS-PAGE and transferred onto PVDF membranes. The membranes were blocked in 3% BSA overnight at 4°C. Blocked membrane were incubated with anti-HA polyclonal antibody (1∶1000; Thermo Fisher Scientific, Waltham, MA) for 1 hour at room temperature. Membranes were washed 3 times for 10 minutes in TBST (0.1%) and incubated with an AP conjugated anti-rabbit secondary antibody (1∶5000; Rockland Inc., Gilbertsville, PA). The ChR2 band was visualized using developing solution (Bio-Rad Laboratories Inc., Waltham, MA).

### Electrophysiology

Microelectrodes were fabricated by pulling borosilicate glass capillaries (World Precision Instruments, Sarasota, Florida) using a PC-10 pipette puller (Narishige, Japan). The microelectrodes had resistances between 0.5–2.0 MΩ. Oocytes were placed in an RC-10 oocyte chamber (Warner Instruments, LLC; Hamden, Connecticut). Solutions were introduced to the chamber using a VC-6 6 channel perfusion valve control system (Warner Instruments LLC; Hamden, Connecticut). Activation of ChR2 was achieved using 75 W xenon arc lamp (Specialty Optical Systems, Inc., Dallas, Texas) and 2 mm light guide (∼4×10^21^ photons s^−1^ m^2^). Currents were measured using a Turbo-Tec 03X amplifier (npi electronic GmbH, Germany) set to voltage clamp mode. The membrane potential was varied from −120 mV to +60 mV in 20 mV steps. Data was recorded using pClamp10 software (Axon Instruments, Inc., Burlingame, California) and further analyzed using Clampfit 10.2 and SigmaPlot. Apparent kinetic values were determined by fitting current traces to a biexponential equation at −120 mV in Na^+^ solution.

### Minimum Pore Estimation

The minimum pore diameter of ChR2 was calculated using excluded volume theory [10], [36]. In brief, the permeability of an ion moving through a channel is proportional to the region left unoccupied by the ion. Plotting the square-root of permeability ratios versus ionic radius, a linear regression is observed according to the equation:
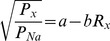
(1a)

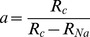
(1b)

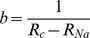
(1c)where R_x_ is the radius of ion X^+^, R_c_ is the radius of the channel, and R_Na_ is the radius of the sodium ion. Calculating 2a/b gives the minimum pore diameter of the channel.

### Structural Modeling of ChR2

Structural models were created using Visual Molecular Dynamics software [37] with PDB entry 3UG9 [29]. Serine residues were inserted into the structure by creating an automatic protein structure file (psf) from 3UG9. Next, the psf was modified using the mutator plugin for VMD to insert serine residues at the homologous positions in the model.

## Results

**Figure 2:**
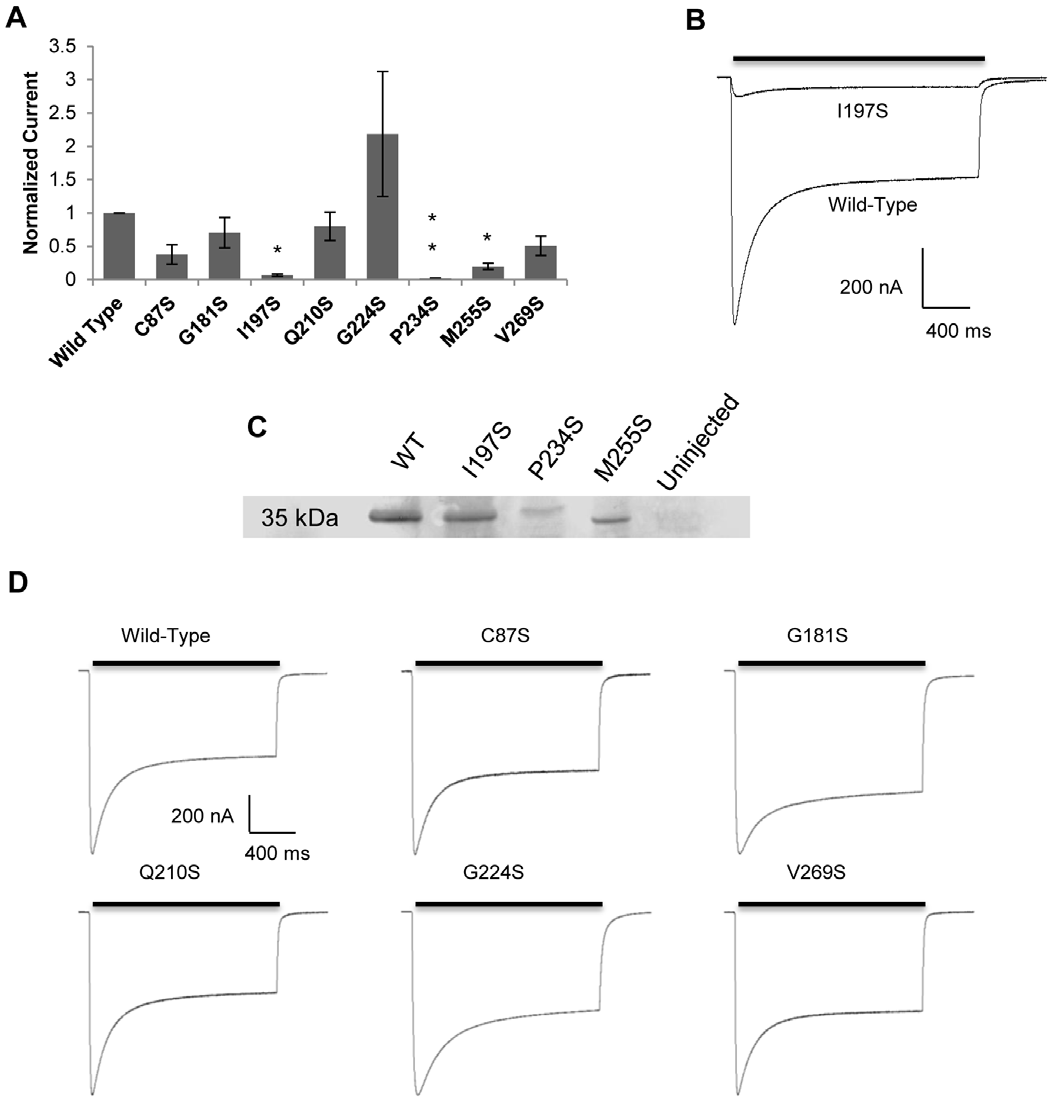
Representative ChR2 photocurrent traces recorded at −120 mV. Black bars indicate illumination with 473 nm light. (A) Normalized photocurrent traces. Mutant photocurrents were normalized to WT ChR2 on the same day in 115 mM Na^+^ solution at pH 9. Values are reported as mean±S.E.M (n = 3–9). Statistically significant values are denoted by a *(*P<0.001; **P<0.04). (B) Reduced photocurrent for I197S compared to WT ChR2 on the same day. M255S and P234S had similar results. (C) Western blot of I197S, P234S, and M255S ChR2 mutants. ChR2-HA tagged band appears at ∼35 kDa. (D) Typical electrophysiological recordings for single serine mutants. Mutants retained inward rectification of cations, but with altered permeability, kinetics, and/or inactivation ratios.

Eight individual serine mutations were made at residues that corresponded to transmembrane serine residues in bR. Mutations were located in various areas of the transmembrane domain; C87S, G181S, G224S, and M255S were located near the retinal binding pocket, I197S, Q210S, and V269S were located at the cytoplasmic end of the transmembrane domain, and P234S was located at the extracellular end of the transmembrane domain (Figure 1B). ChR2 constructs were expressed in *Xenopus laevis* oocytes and photocurrents were recorded using two-electrode voltage clamp techniques. Photocurrent measurements were made in the presence of 115 mM alkali metal solution (115 mM XCl, 2 mM BaCl_2_, 1 mM MgCl_2_, Tris; pH 9). Function of mutant constructs was determined by measuring photocurrents at −120 mV in 115 mM Na^+^ solution and compared to WT ChR2 photocurrents on the same day (Figure 2A). The mutants I197S, P234S, and M255S were functional, but had highly reduced photocurrents (Figure 2B). Surface expression of I197S, P234S, and M255S was determined by Western blotting (Figure 2C). Both I197S and M255S have similar band intensity compared to WT ChR2, suggesting that these mutations have reduced functionality. Conversely, P234S showed very low expression compared to the wild-type. However, because of the low photocurrents I197S, P234S, and M255S were not analyzed further. The five mutants that were measured retained typical inwardly rectified behavior (Figure 2D).

**Figure 3:**
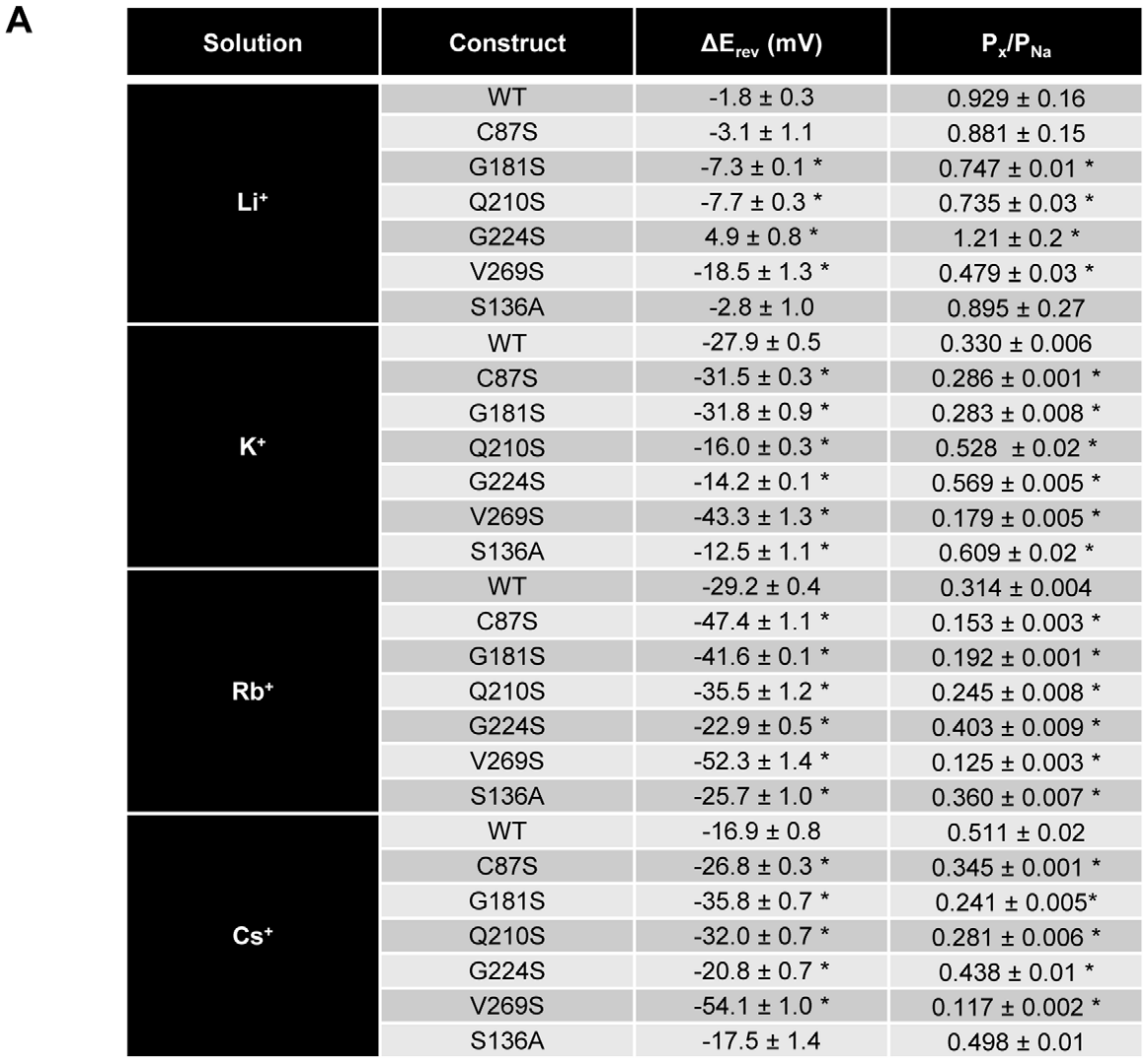
Shifts in reversal potential and permeability ratio comparison of single serine ChR2 constructs. Changes in reversal potential were calculated by subtracting E_rev_ in cation X^+^ from E_rev_ in Na^+^ (mean**±**S.E.M; n = 7–25). Permeability ratios were calculated using changes in reversal potential. Values are reported as mean±S.E.M (n = 7–25). For significance testing, mutant ChR2 values were compared to WT in the same solution. Statistically significant values are denoted by a *(P<0.001).

To measure permeability ratios of mutant ChR2 constructs, we measured shifts in reversal potential using Li^+^, K^+^, Rb^+^, and Cs^+^ solutions (pH = 9) (Figure 3) relative to Na^+^ for individual constructs. Typical electrophysiological recordings for each mutant are shown in Figure 2D. Mutants retained the inwardly rectified current of WT ChR2 in each alkali metal solution. Photocurrents were normalized to the largest signal in Na^+^ solution on the same day for E_rev_ analysis. Large negative changes in E_rev_ were observed for C87S (ΔE_rev_ = −47±1 mV; Rb^+^) and V269S (ΔE_rev_ = −52±1 mV; Rb^+^) in the presence of larger cations when compared to WT ΔE_rev_ values (ΔE_rev_ = −29.2±0.4 mV; Rb^+^). A negative shift in reversal potential indicates that the channel becomes less permeable to cation X^+^. In contrast, G224S had much smaller shifts in E_rev_ (ΔE_rev_ = −14.2±0.1 mV; K^+^) which indicated alkali metals became more permeable relative to other constructs and the phenotype (ΔE_rev_ = −27.9±0.5 mV; K^+^). Both G181S and Q210S had mixed results, with both larger and smaller shifts in E_rev_ compared to WT ChR2 (Figure 3). Interestingly, three mutants (G87S, G181S, and V269S) had highly positive sodium E_rev_ indicating an increased selectivity for this ion over the other alkali metals. Other mutants had Na^+^ E_rev_ similar to wild type.

Next, we used ΔE_rev_ to calculate relative permeability ratios, P_x_/P_Na_, of each ion for ChR2 mutants. Changes in E_rev_ between X^+^ and Na^+^ for each mutant were used to exclude differences in the cytoplasmic ionic composition of oocytes. Ratios were calculated relative to 115 mM Na^+^ solution for each ChR2 mutant (Figure 3). Values>1 indicated a higher permeability for cation X^+^ over Na^+^, where values<1 indicated reduced permeability for cation X^+^. In general, the largest changes in permeability ratios were seen for the larger cations (K^+^, Rb^+^, and Cs^+^). V269S showed highly reduced permeability ratios when compared to WT in all solutions. In contrast, G224S had higher permeability ratios when compared to WT except for P_Cs_/P_Na_, where it was comparable to WT.

**Figure 4:**
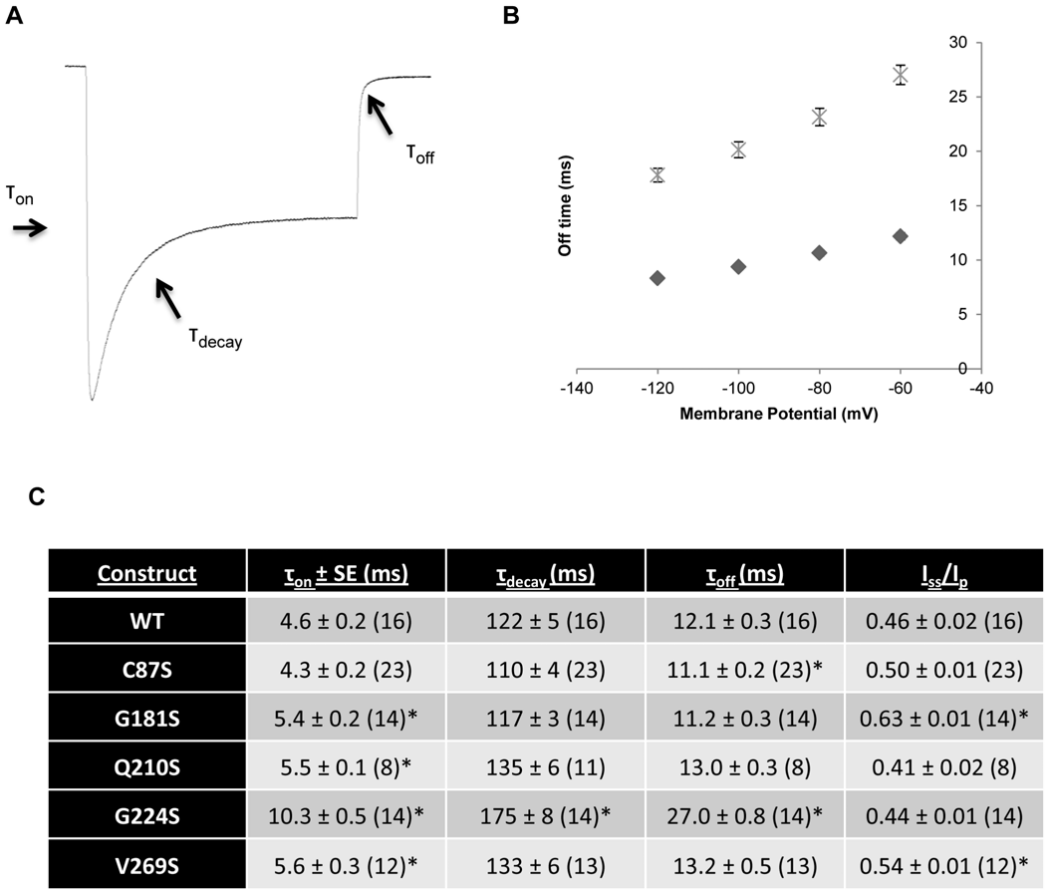
Kinetic parameters for single serine ChR2 constructs. Kinetic values were obtained by fitting ChR2 photocurrents to a standard biexponential equation. All parameters were calculated in 115 mM Na^+^ solution at pH 9. For on and decay times, traces were fit at −120 mV to ensure ChR2 was in the dark adapted state. Values reported as mean±S.E.M (n = 8–23). Significant differences are marked with a *(P<0.05). (A) Summary of photocurrent exponential fits. On time was calculated from baseline current to peak current, decay time was calculated from peak to stationary current, and off time was calculated from stationary to baseline current when the light is switched off. (B) Off rate comparison of the slow G224S mutant versus WT at different membrane potentials. The off rate of ChR2 had a strong dependence on the holding potential of the cell. Diamond, WT; crossed X, G224S. All values were statistically significant (as described in C) (C) Summary of kinetic parameters and inactivation ratios for single serine ChR2 mutants. Values are reported as mean±S.E.M (n = 8–23). Statistically significant values are denoted by a *(P<0.05).

We measured kinetics and steady-state to peak current ratios (I_ss_/I_p_) to determine the effect of single serine mutations on the ChR2 photocycle. Current traces were fit in Na^+^ solution from the dark to peak region for on rates (τ_on_), the peak to steady-state region for decay rates (τ_decay_), and the steady state to dark region for closing rates (τ_off_) (Figure 4A). Additionally, opening and decay rates were fit at −120 mV to ensure ChR2 was fully recovered. WT ChR2 had an off rate of 12.1±0.3 ms (−60 mV), on rate of 4.6±0.2 ms, and decay rate of 122±5 ms (n = 16) (Figure 4B and 4C). Each mutant ChR2 had similar results for channel closing except for G224S which showed a two-fold increase in channel closing (27.0±0.8 ms; n = 14; −60 mV) (Figure 4B). Decay rates for ChR2 mutants demonstrated a similar trend with the off rates (Figure 4C). Only G224S was slower with a decay rate of 175±8 ms (n = 14). Single serine ChR2 on rates were statistically significant when compared to wild-type, except for C87S which had no change. G224S had the largest change in on rate with τ_on_ = 10.3±0.8 ms (n = 14). Q210S also showed a slight slowdown with τ_on_ = 5.5±0.1 ms (n = 8) (Figure 4C). G181S and V269S had τ_on_ values of 5.4±0.2 ms and 5.6±0.3 ms, respectively. Our kinetic data show that only G224S exhibited a global effect on the ChR2 photocycle and that several ChR2 mutants selectively increased the opening channel kinetics.

**Figure 5:**
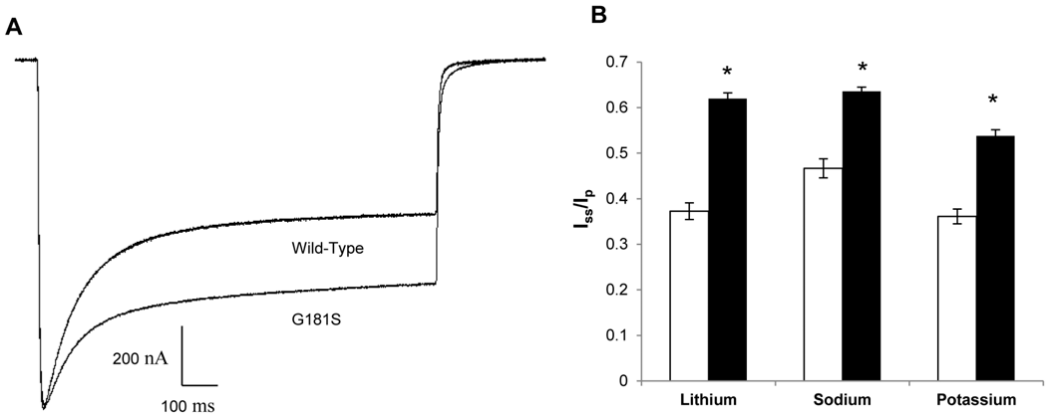
Steady-state to peak current ratios for WT and G181S ChR2. Ratios were calculated after normalization to −1 µA at −120 mV in cationic solutions (115 mM XCl, 2 mM BaCl_2_, 1 mM MgCl_2_, Tris; pH 9; n = 14–18). Significant differences are marked with a *(P<0.05). (A) Comparison of WT and G181S photocurrents in Na^+^ solution. (B) I_ss_/I_p_ comparison for WT and G181S in different cationic solutions. Wild-type ChR2 is colored white while G181S is colored black.

Steady-state to peak current ratios were also calculated for each mutant ChR2. Ratios were calculated in Li^+^, Na^+^, and K^+^ solutions by normalizing current traces at −120 mV to −1 µA. I_ss_/I_p_ ratios are used as a measure of inactivation of the channel during prolonged light exposure. Additionally, because monovalent cation selectivity decreases in tandem with an increase in proton selectivity during the steady state, these ratios can be used to infer increased cation selectivity in the absence of protons (pH = 9) [14]. G181S had larger ratios when compared to WT in the same solution, which indicated less inactivation as ChR2 transitions to the O2 state (Figures 1A and 5). Other ChR2 mutants had no significant difference or a change of I_ss_/I_p_ in only one solution (Figure 4C).

**Figure 6:**
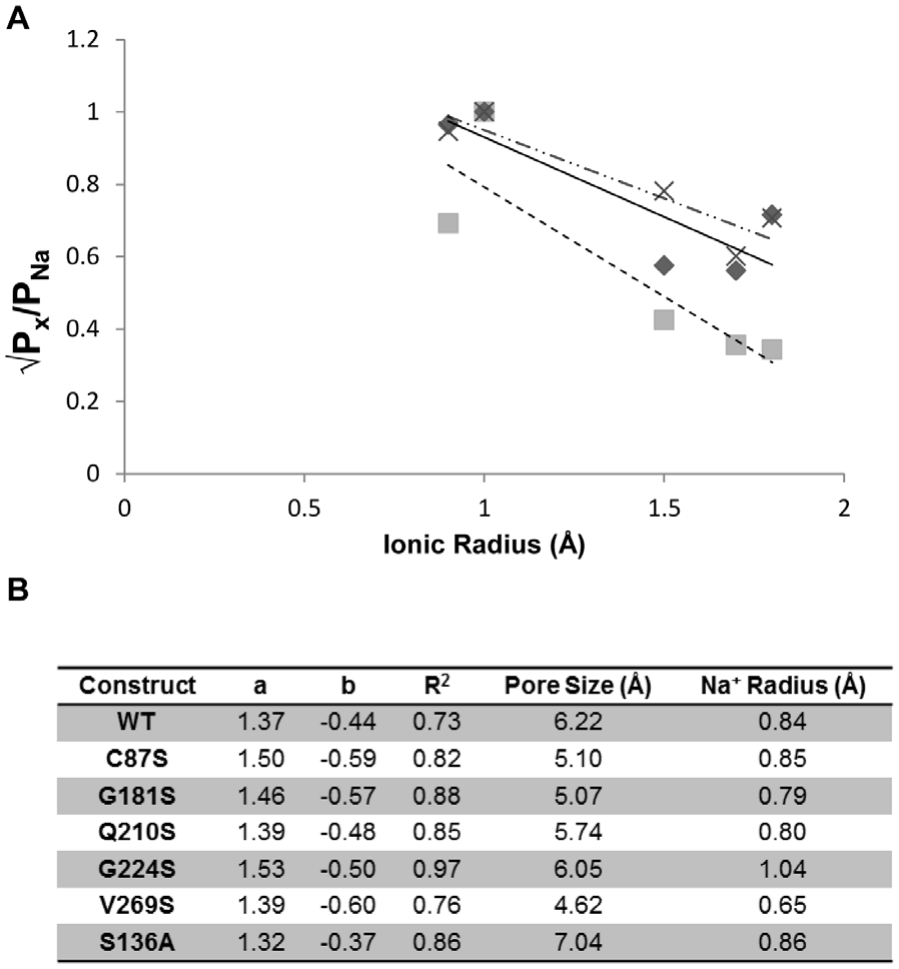
Relationship of relative permeability and alkali ionic radii for ChR2 serine mutations. Relative permeability ratios were calculated using reversal potentials for each mutant. (A) Plotting the square root of relative permeability ratios vs. ionic radius yielded a linear fit for each data set. Solid line/diamond, WT; dashed line/square, V269S; broken line/X S136A. For all mutants, the larger alkali metal ions (K^+^, Rb^+^, and Cs^+^) were less permeable than the smaller alkali metal ions (Li^+^, Na^+^). These results were consistent with excluded volume theory [36]. (B) Minimum pore fitting parameters and pore size calcualtions for single serine ChR2 mutants and S136A. The coefficients a and b correspond to the y-intercept and slope of the fitted data, respectively.

To determine whether introducing serine residues into the transmembrane domain of ChR2 facilitates inter- and intrahelical hydrogen-bonding, we estimated the minimum pore diameter of each construct. We applied excluded volume theory using our calculated permeability ratios to estimate the narrowest region of the channel. The permeability of an ion permeating the channel is proportional to the region that is left unoccupied by the ion in the pore [10], [36], [38]. Thus, the diameter of the narrowest region of the pore can be determined by plotting the square root of permeability ratios P_x_/P_Na_ versus ionic radii. Values of the y-intercept, a, and slope, b, were obtained by fitting the data with a linear regression (Figure 6). Fitting the square root of WT permeability ratios to a linear regression afforded values of 1.37 and −0.44 for a and b, respectively with an R^2^ value of 0.73. R^2^ values were consistent with previously published pore size calculations [36]. According to equation 1a, the minimum pore diameter for WT ChR2 was 6.22 Å, in good agreement with the previously described estimates [4]. Single serine mutations had calculated minimum pore sizes smaller than that of WT ChR2, ranging from 4.62 Å to 5.74 Å (Figure 6B) excluding G224S, which had a pore size of 6.02 Å. The largest change in pore diameter was seen for V269S which had a pore size of 4.62 Å. The change in pore size was consistent with our permeability calculations for this mutant ChR2, where all cations had decreased P_x_/P_Na_. Calculating (1–a)/b (Equations 1b and 1c) gives the radius of a sodium ion permeating the channel. The value obtained for WT was 0.84 Å (Figure 6B). Calculated sodium radii were found to have ∼15% error compared to the dehydrated radius of sodium (0.98 Å) and within reason compared to previously published reports [10], [39], [40].

Lastly, we replaced an endogenous serine residue located at position 136 with alanine in order to remove an inter- or intrahelical hydrogen bond. We hypothesized that this would cause an opposite effect on ChR2 by increasing the pore diameter. Using permeability ratios (Figure 3) and applying excluded volume theory, we determined the minimum pore diameter to be 7.04 Å with an estimated sodium radius of 0.86 Å (Figure 6). Removal of S136 provides further evidence that introduction of transmembrane serine residues constrict the pore diameter through inter- or intrahelical hydrogen bonds.

## Discussion

ChR2 has become a popular tool in the neurosciences for the temporal and spatial activation of excitable cells with light [41]–[44]. However, the full mechanism for ion conductance is not well understood. Furthermore, why ChR2 is an ion channel when it shares a high sequence homology with other microbial-type rhodopsins, which are ion pumps, continues to be an unanswered question. Previous experiments utilizing FT-IR difference spectra show that ChR2 undergoes large conformational changes in its backbone structure compared to bR [18]. A sequence analysis of these two proteins indicates ChR2 also lacks eight transmembrane serine residues present in bR. Considering that serine residues within transmembrane domains contribute to inter- and intrahelical hydrogen-bonding, we hypothesized that the lack of these serine residues were an important contributor to the molecular determinants that define ChR2 function. By reintroducing the eight transmembrane serine residues present in bR at homologous positions in ChR2, we have shown that the pore size is decreased. Also, replacement of S136 with alanine increased the pore diameter. An analysis of the crystal structure of the channelrhodopsin chimera C1C2 (PDB entry: 3UG9) indicates that some serine mutations have the potential to form hydrogen-bonds with residues on opposite helices or with the carbonyl backbone. Serine residues were inserted into the crystal structure using the mutator plugin for VMD.

Three ChR2 mutants, I197S, P234S, and M255S, showed little ion conductance upon activation with light. The expression of both I197S and M255S were similar to WT, suggesting that these two mutations cause decreased function. In contrast, the P234S had highly reduced expression compared to WT. The reduced photocurrent is therefore a consequence of expression and not an alteration to the channel.

The most drastic change in pore diameter was observed for the V269S mutant, which had permeability ratios smaller than WT ChR2. V269S resides on the intracellular end of TM 7 near the transmembrane and intracellular interface. This helix extends past the membrane ∼18 Å into the cytosol [29]. The closest hydrogen-bonding acceptor is E273, where the carboxylate ion is ∼3.78 Å away from the hydroxyl proton on V269S. V269S is also in close proximity to the intracellular ends of TM1 and TM2, but the structure is unresolved at the residues of interest (residues 80–88). Although there are no explicit hydrogen-bonding partners closer than 3.78 Å, the exposure of this residue to the cytosolic fluid may facilitate a water-mediated hydrogen bonding network that holds TM1 in place during the photocycle. It has been suggested that TM1 may form the pore exit during the photocycle [29]. Additionally, it has been previously shown for the M intermediate in bR that S226 (V269S in ChR2) moves ∼2 Å closer to L100 (T139 in ChR2) [45]. This region has also been shown to form a hydrophobic pocket that contains a water molecule, which could form a hydrogen-bonded network between V269S and T139 [46], [47]. We postulate that locking this helix into place through a water-facilitated hydrogen-bonding network causes the decrease in pore size for this ChR2 mutant.

The C87S and G181S mutants also had large reductions in pore size (5.10 Å and 5.07 Å, respectively). G181S is located next to the β-ionone ring of the retinal moiety (∼2.36 Å), but has no effect on the kinetics of the photocycle. However, G181S is 2.08 Å from the backbone carbonyl of T159 and 3.61 Å from the hydroxyl group of T159, which provides two possible hydrogen-bonding sites. This threonine residue has been shown to form part of the retinal binding pocket where it is important for photocycle kinetics, magnitude of photocurrent, and the light intensity required for activation [48]. Furthermore, we observed an increase in steady-state to peak current ratios for G181S under continuous light conditions, which suggests that this residue has an indirect effect on ChR2 inactivation. This is most likely a result of an interaction with T159. The C87S mutant has one possible interaction with the backbone carbonyl of S63 at a distance of ∼2.32 Å. This region of the pore is constricted through hydrogen-bonding of S63, E123 and N258. The introduction of a hydrogen-bond with C87S would further constrict this region by coupling TM1 and TM2. Interestingly, cysteine residues also have the propensity toward forming hydrogen-bonds similar to serine in membrane protein structures [49].

Both Q210S and G224S had small changes in the pore diameter. Analysis of the channelrhodopsin chimera crystal structure suggests that Q210S is oriented toward the lipid bilayer where it has no potential for hydrogen bonding to the channel backbone. This explains why only a small change in pore diameter was observed for this mutation. Lastly, G224S had nearly no change in pore diameter compared to WT ChR2. The side chain, similar to Q210S, is oriented into the bilayer. There is potential for a hydrogen-bond to be formed to the carbonyl of F220, although the distance is 3.91 Å; above the average for this type of interaction. The changes in kinetics may be explained by having a glycine at this position in the wild-type. G224 could facilitate a favorable helix-helix interaction during the photocycle. Addition of a hydrogen-bond would remove this interaction but the added rigidity of the protein would cause slower kinetics and a pore diameter consistent with the phenotype.

To further test our hypothesis that serine residues play an important role in the conformational flexibility of ChR2, we replaced a serine at position 136 with alanine. The results from experiments with S136A indicated a pore size larger than that of WT ChR2, which was the opposite effect that we observed when introducing serine residues into the transmembrane domain. S136 resides on TM4, approximately 3.2 Å away from T149 on TM5, which provides a potential hydrogen bonding site. Mutation of S136 to alanine would remove this interaction and thus allow for pore expansion. These results suggest that the reduced pore diameter of single serine ChR2 mutants is caused by the introduction of a serine mediated hydrogen-bond.

We calculated permeability ratios for the five single serine insertions and a single serine removal as a measure of channel selectivity to further support the notion that introduction of hydrogen-bonds would restrict the conformational flexibility of the channel. In general, ChR2 mutants that decreased the size of the pore had reduced relative permeability ratios for alkali metals. The V269S mutant had the largest effect on permeability, which was expected because of the large change in pore diameter. C87S, G181S, and Q210S had permeability ratios smaller than wild type for most ions, but larger than V269S. G181S also was the only mutant ChR2 that had a change in steady-state to peak current ratios compared to WT ChR2. An analysis of our experimental results suggest that the size selectivity filter is not restricted to any specific location within the transmembrane domain, as these residues are located on different helices either at the intracellular end or near the retinal binding pocket. Furthermore, the G224S mutant was the only construct that had a change in the apparent kinetic parameters of the channel. The slowdown in kinetics we observed was a global effect which persisted throughout the entire conducting state. Excluding G224S, serine mutants had little effect on apparent channel kinetics. This suggests that pore constriction is not a result of a global change in the protein, but rather a local effect arising from the introduction of a strong hydrogen bond donor.

Calculation of the pore size can be determined both empirically and computationally [10], [50]. Our approach used excluded volume theory, which has general applicability to the translocation of hydrated and dehydrated cations [31], [34], [36]. This method has been well established for accurate estimations of the minimum pore size for ion permeation [10], [36]. It is important to note that pore calculations are determined using relative permeability ratios. Therefore, it is possible to see a net decrease in the permeability of all cations with no change in pore diameter. Likewise, a proportional increase in cation permeability would result in no change in pore size. An example of this is the G224S mutant.

Recently, Nack *et al.* reported that 0.3 H^+^/ChR2 are pumped during the photocycle, a contribution that we have not taken into account for pore size estimations [51]. However, the amount of protons pumped during the photocycle is small in comparison to the amount of ions flowing through the channel. Therefore, we believe that the proton pumping ability of ChR2 has a negligible effect on the pore size calculations.

In summary, the recently elucidated crystal structure of the chimeric channelrhodopsin protein (C1C2) exhibited a large pore lining surface on the extracellular side of C1C2 [29]. The putative conducting pathway forms between transmembranes 1, 2, 3, and 7 and is lined with negatively charged residues. Our results suggest that the size selectivity filter is located further along the channel and at multiple points along the conductance pathway (Figure 1B). Furthermore, evidence has been presented that residues located adjacent to the retinal binding pocket, along the intracellular side of the protein, and an interaction between TM1 and TM2 all affect the permeability of ChR2. Thus, residues that are located on the cytoplasmic side of the channel are determinants of ion conductance. We also suggest that the high affinity for serine residues to form inter- and intrahelical hydrogen bonds is the cause for reduction in pore size, decrease in permeability ratios, and slowdown of kinetics. The channel activity of ChR2 appears to be a direct consequence of its ability to undergo large conformational changes in the backbone structure for pore formation.
